# Factors associated with health service orientation and active product marketing orientation in Finnish community pharmacies: a nationwide study among private pharmacy owners

**DOI:** 10.1186/s12913-020-05469-y

**Published:** 2020-07-20

**Authors:** Lenita Jokinen, Inka Puumalainen, Marja Airaksinen

**Affiliations:** 1 Runosmäki Pharmacy, Turku, Finland; 2 University Pharmacy, Helsinki, Finland; 3 Clinical Pharmacy Group, Division of Pharmacology and Pharmacotherapy, Faculty of Pharmacy, University of Helsinki, Helsinki, Finland

**Keywords:** Community pharmacies, Strategy, Pharmacy services, Healthcare, Product marketing

## Abstract

**Background:**

Little is known about pharmacy owners’ commitment to public health and health policy goals in the strategic planning of their business. The aim of this study was to explore factors associated with health service orientation and active product marketing orientation of Finnish community pharmacy owners.

**Methods:**

A national cross-sectional e-mail survey was sent to private community pharmacy owners in Finland (*n* = 581) in 2013. Based on the structured, Likert-type survey instrument, two sum scales measuring strategic orientation towards health service provision (13 items, score range 0–26) and active product marketing (8 items, score range 0–16) were developed (Cronbach’s Alpha 0.836 and 0.699, respectively). Characteristics of the pharmacy owners and their pharmacy business as well as actual service provision were used as background variables.

**Results:**

Concerning health service orientation, 50% of the respondents received at least 20 points out of the maximum 26 points (score range: 0–26). For active product marketing orientation, 75% of the pharmacy owners had at least 14 points and 44% received full 16 points (score range: 0–16). The score distribution was skewed towards strong health service orientation, but the actual service score was heavily skewed towards few services or no services. Two-thirds of the pharmacy owners reported having available 2 or less services. The health service orientation was not influenced by any of the background variables used, but three of them influenced active product marketing orientation, namely business location, annual prescription volume and belonging to a marketing chain of individual community pharmacies.

**Conclusion:**

Large pharmacies located close to rivals and belonging to marketing chains of individual community pharmacies differentiated as those having a high product marketing orientation. The health service orientation was not influenced by any of the explanatory variables used in this study. The discrepancy between high health service orientation scores and low actual service provision scores needs further investigation. The contradiction that exists between pharmaceutical policy goals and the generation of income of pharmacies should also be examined as a contributing factor in this respect.

## Background

The marketplace of community pharmacies is undergoing remarkable changes in many countries throughout the world [1–4]. This can be due to major societal, financial and political changes which can lead to major changes in social and health services systems. A dominating political trend has been towards deregulation of pharmacy ownership and non-prescription medicine sales [4]. The main arguments favoring deregulation have been related to better access to pharmacies and pharmaceuticals in terms of number of outlets, opening hours, location and prices of medicines [5, 6]. On the other hand, there has been a demand for pharmacies to extend their services towards health care to support rational and safe use of medicines. This trend towards health services has been strongly supported by the pharmacy profession (e.g., FIP/WHO 2011), though it has been challenging to communicate it to policy makers and consumers, despite the fact that consumers trust pharmacists and are satisfied with their customer service [3, 7, 8]. However, consumers are not aware of the health service’s pharmacists offer but regard them as traditional medicine distributors and dispensers [1, 2, 9, 10].

Community pharmacy owners as private entrepreneurs decide on the strategic development of their business. However, it is not well known how committed the pharmacy owners are to public health and health policy goals in the strategic planning of their business [10]. There exists previous research on how the earnings model of pharmacies has been reformed in some countries [11, 12]. The aim has been to move to a service-based earnings model that would encourage pharmacists to provide services to health care to support rational and safe use of medicines [11, 12].

The community pharmacy system in Finland consists of approximately 800 privately owned outlets, of which about 600 are main pharmacies and 200 subsidiary outlets [13]. In addition to these, there are two university-owned community pharmacies with 18 outlets in different parts of Finland. The number of outlets has remained quite the same throughout the years. Ownership of a pharmacy is granted via a license from the National Agency for Medicines. Pharmacy chains are not allowed, but pharmacy owners can operate up to three subsidiaries, smaller service points with a limited assortment and online pharmacies that can sell prescription and non-prescription medicines. Pharmacy owners have established marketing chains to gain purchasing power and marketing volume, as well as jointly organize personnel training. A majority (80%) of the pharmacies´ market share consists of prescription drug sales, while the rest consists of non-prescription drug sales (14%) and sales of other health-related products (6%) [13]. Finnish community pharmacies pay an additional government fee, a “pharmacy tax”, which is progressively calculated as a proportion of annual turnover (range 1–11%, mean 7%) [13]. The purpose of the pharmacy tax is to offset the profits of pharmacies, but the tax does not compensate the large income gap between the pharmacies with low and high turnover.

The structure of the Finnish community pharmacy system has remained quite the same throughout the last decades, compared to the structural changes towards liberalization within other Nordic and European Union countries [4, 6]. Previous and current medicines policy documents have strategically integrated community pharmacies into the social and healthcare system [14–17]. The same tendency can be seen in the most recent policy documents outlining community pharmacies’ tasks and functions in the major healthcare reform currently under way in Finland [15].

We conducted a national survey in Finland, in 2013, involving all private community pharmacy owners in order to assess whether having a strategy influenced their health service and active product marketing orientation [18]. It was found that not all pharmacy owners had a strategy, but their strategic vision by 2020 concerning health services orientation was the same regardless of whether they had a strategy or not. Having a strategy was associated with variables indicating active product marketing orientation, such as whether the pharmacy had a person responsible for marketing campaigns and actively invested in add-on-sales of non-prescription medicines and non-medical products. Pharmacy owners running their outlets in competitive marketplaces, such as in a city or commercial center, were most likely to have a strategy. This present study continued investigating the same theme concerning private pharmacy owners’ strategic orientation towards health services and active product marketing. The specific aim of this study was to explore factors associated with health service orientation and active product marketing orientation of Finnish community pharmacy owners.

## Methods

### Study design

This study was conducted by using data derived from a larger national cross-sectional e-mail survey sent to private community pharmacy owners in Finland, investigating their strategy work related to running their community pharmacy business [18]. The survey covered all members of the Association of Finnish Pharmacies (*n* = 581 at the time the survey was conducted in March 2013), forming a majority of the approximately 600 private community pharmacy owners in Finland. The details regarding conducting the survey as well as the development of the survey instrument have been reported elsewhere [18].

### Measures used in this study and statistical analysis

The entire survey instrument is presented in Additional File 1. The survey instrument primarily consisted of structured questions applying a 5-point Likert-scale format, with the options ranging from fully agree to fully disagree. The questions were divided into five sections: characteristics of the pharmacy owner, characteristics of the pharmacy’s strategic development, marketing efforts, and range of services provided in the pharmacy at the time of the survey and plans on investing in health care-oriented service development by 2020.

In our previous study, the data were divided into dichotomies based on whether the pharmacy had a strategy or not [18]. Thus, having a strategy was the dependent variable. Among the explanatory variables were two sum scales that were constructed to describe health services orientation and active product marketing orientation [18]. These sum scales were used as dependent dichotomous variables (strong or weak orientation) in the present study. The explanatory variables included characteristics of the pharmacy owner, such as work experience as a pharmacy owner and gender; characteristics of the pharmacy business, such as pharmacy’s location by province and location area (e.g., countryside, city center), annual prescription volume and turnover. Other explanatory variables used were: 1) whether the pharmacy owner had a strategy for running his/her pharmacy business; 2) whether he/she had plans to resign/retire, or to apply for a license for another pharmacy within 3 years (if the new license is granted by the Medicines Agency, the owner needs to terminate their existing license); 3) whether the pharmacy belonged to a marketing chain of individual community pharmacies; and 4) the range of actual services available in their pharmacy at the time of the study in 2013, presented as a summative variable.

The summative variable measuring health service orientation was developed by combining 13 independent Likert-scale variables related to health service orientation of Finnish community pharmacies at the time of the survey (Additional File 2, Appendix 1). The same kind of summative variable was calculated for eight independent variables related to active product marketing to operationalize active product marketing orientation (Additional File 2, Appendix 1). The internal consistency of both summative variables was calculated by using reliability analysis (Cronbach’s Alpha).

Responses were scored so that the fully agree and agree to some extent responses yielded 2 points while other responses yielded 0 points. The score range for 13 variables measuring health service orientation was 0–26, and for 8 variables measuring active product marketing orientation was 0–16. Responses to both summative variables were divided into two dichotomous categories to indicate 1) strong or weak health services orientation (strong orientation: scores 20–26; weak orientation: scores 0–18) and 2) strong or weak active product marketing orientation (strong orientation: scores 14–16; weak orientation: scores 0–12). The division into strong and weak orientation was made by halving the frequencies. A bivariate analysis was performed to explore the association between background variables, i.e., explanatory variables, health service orientation and active product marketing orientation. Values of *p* < 0.05 were considered statistically significant.

The pharmacy owners were also asked to report the range of actual services available in their pharmacy at the time of the study in 2013. For the analysis, a summative variable was calculated based on the services available: each service equaled one point. Thus, the scale range was 0–10. The correlations of the summative variable reflecting actual service provision with active product marketing orientation and with health services orientation was calculated. Appendix 2 provides a more detailed description of the health-related services included in this study that were available in Finnish community pharmacies or part of the pharmacies at the time of the survey in 2013.

Responses were analyzed using the SPSS program (version 22.0). The results are presented as frequency distributions and cross tabulations. The statistical significance was tested using the Chi Square test (*p*-value of < 0.05 was considered statistically significant). The correlations between the variables were calculated by Pearsons’s correlation test.

## Results

The response rate was 34% (*n* = 198). Of the respondents, 42% were experienced pharmacy owners who had owned a pharmacy for more than 10 years. The majority (72%) of the respondents were women. The gender distribution and their pharmacy prescription volumes represented pharmacies in Finland well. The respondents represented evenly pharmacies of different sizes in terms of annual turnover and prescription volume. Of the respondents 45% operated their pharmacy in a rural community, and 41% in a location with rivals (22% in city centers and 19% in city commercial centers such as shopping malls). About one-third (38%) of the pharmacies belonged to a marketing chain. Almost two-thirds (63%) reported that their pharmacy had a strategy. Of the respondents, 40% planned to retire or apply for a license for a new pharmacy from the Finnish Medicines Agency within a 3-year period after the time of the survey. More detailed background information has been reported elsewhere [18].

**Fig. 1:**
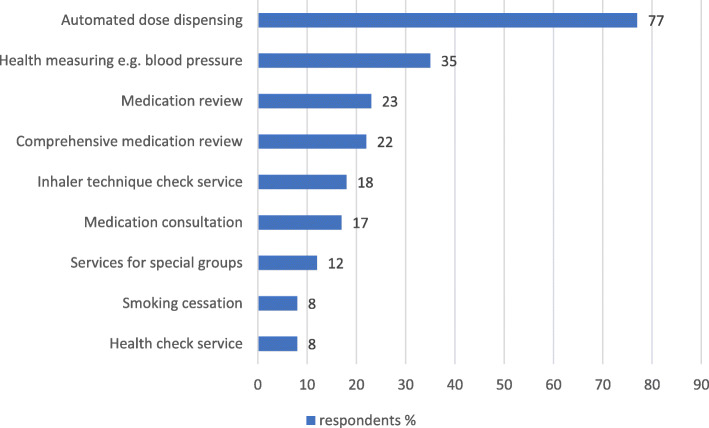
Provision of different health-oriented services in community pharmacies at the time of the survey in 2013 (% of the responding pharmacy owners, *n* = 188–196; the number of actual respondents is marked for each service; respondents could identify as many services as applicable, see Additional file 2: Appendix 2 for more detailed description of the health-related services included in the study)

Automated dose dispensing was the most common health-oriented service provided by most of the responding pharmacies (77%) (Fig. 1). Other health-oriented services were provided by less than half of the pharmacies: health measuring was the most provided (35%) while health check service was the least provided (8%).

**Fig. 2:**
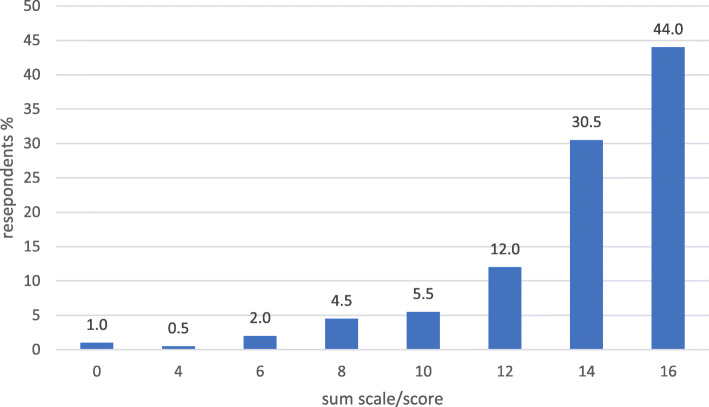
Pharmacies’ active product marketing orientation as measured by a sum scale consisting of 8 variables (% of the responding pharmacy owners, *n* = 198; the range of the sum scale 0–16, see Additional file 2: Appendix 1 for more detailed description of the sum scale)

**Fig. 3:**
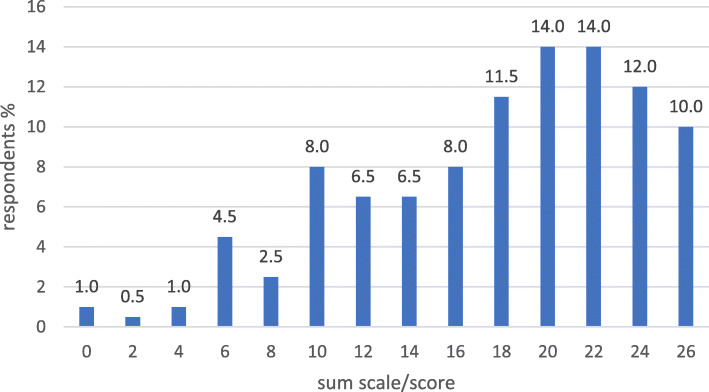
Pharmacies’ health services orientation as measured by a sum scale consisting of 13 variables (% of the responding pharmacy owners, *n* = 198; the range of the sum scale 0–26, see Additional file 2: Appendix 1 for more detailed description of the sum scale)

**Fig. 4:**
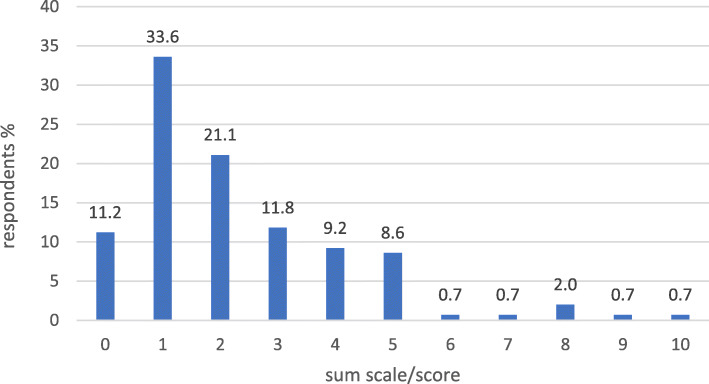
The summative presentation of health-oriented services actually provided at the time of the survey in 2013 (each available service yielded 1 point, score range 0–10, see Additional file 2: Appendix 2 for more detailed description of the health-related services included in the study)

The internal consistency for the set of 8 variables indicating a pharmacy’s active product marketing orientation was 0.699 (Cronbach’s Alpha), and for the 13 variables indicating pharmacy’s health service orientation it was 0.836. Concerning active product marketing orientation, 44% of the pharmacies received a full 16 points and 75% at least 14 points (Fig. 2). The score range was more scattered for health services orientation, although 50% of the respondents received at least 20 points out of the maximum 26 points (Fig. 3). The summative score presentation of the health-oriented services provided at the time of the survey in 2013 was controversial, as 66% of the pharmacy owners reported having two or less services out of the ten listed in the survey instrument available (Fig. 4).

None of the background variables influenced the health service orientation score (Chi Square test), while three out of eight variables were found to have statistically significant association with active product marketing orientation (Table 1). These variables were pharmacy location (*p* = 0.041), prescription volume (*p* = 0.047) and operating as a part of a pharmacy chain (*p* < 0.001). No correlation was found between actual service provision and health service orientation (Pearson correlation 0.098). There was positive correlation between actual service provision and active product marketing orientation (Pearson correlation 0.503).

**Table 1:** Association between pharmacy location area, annual prescription volume, belonging to a marketing chain of individual private pharmacies and active product marketing orientation (sum scale)

Active product marketing orientation (sum scale)	Low (score 0–12)		High (score 14–16)		Total	***P***-value
	%	n	%	n	n	
**Business location area**						*p* = 0.041
Countryside	62	55	38	34	89	
City, center	64	27	36	15	42	
City, suburb	52	14	48	13	27	
City, commercial center (e.g., shopping mall)	37	14	63	24	38	
Total	56	110	44	86	196	
**Annual prescription volume**						*p* = 0.047
Less than 40,000	71	37	29	15	52	
40,000–600,000	43	16	57	21	37	
60,001–100,000	55	35	45	29	64	
More than 100,000	50	22	50	22	44	
Total	56	110	44	87	197	
**Whether the pharmacy belongs to a marketing chain of individual private pharmacies**						*p* < 0.001
No	72	59	28	23	82	
Yes	43	48	57	63	111	
Total	55	107	45	86	193	

## Discussion

Despite the international and national discussion over the roles and activities of community pharmacies, their societal status remains undefined between commercial and public health functions. Our study is among few published studies that have examined this issue from the perspective of individual pharmacy owners who bear financial responsibility for their business. Research and inventories have begun to emerge on the remuneration and effectiveness, even cost-effectiveness, of pharmacy services, but a large-scale breakthrough in healthcare-oriented pharmacy services is still missing [19–21].

Our results indicate that the health service orientation of Finnish community pharmacies was not influenced by any of the background variables used in this study, covering a wide range of factors crucial for determining pharmacy business in Finland. On the other hand, three of the factors influenced active product marketing orientation, namely business location, annual prescription volume and belonging to a marketing chain of individual community pharmacies. The results also showed a discrepancy between health service orientation and the services provided: the score distribution was skewed towards strong health service orientation, but the actual service score was heavily skewed towards few services or no services. Two-thirds of the responding pharmacy owners reported having two or less services out of the ten listed in the survey instrument available. The actual situation may have been even less favorable for health service provision because only about one-third of all community pharmacy owners responded to our survey. Among non-respondents may have been many pharmacy owners less interested in professional service development and less committed to medicines policy goals favoring integration of community pharmacies in healthcare. This assumption is confirmed by evidence from other studies on community pharmacy services: they are consistent with the finding that a minority of community pharmacists actively provide healthcare-oriented services other than medication counseling [12].

Pharmacies located in shopping centers were most active in product marketing, while pharmacies located in rural communities and city centers were the least active. This may be explained by the fact that pharmacies in rural communities do not have local competitors, so they do not need to compete for customers and market share in the same way as urban pharmacies. We also found that active marketing was not as common in small pharmacies than it was in middle-sized and large pharmacies, as measured by turnover and prescription volume. All these results were relatively expected, as was the finding that belonging to a marketing chain of individual community pharmacies was associated to a more active product marketing. A part of Finnish community pharmacies belong to a marketing chain. The marketing chain, at its best, could facilitate investment in and implementation of health-oriented services as individual pharmacies may not have enough resources to produce and market the services alone [22]. However, traditional chain pharmacies have been strongly oriented to active product marketing which can be seen in many countries where these chains exist [10, 11]. Even in high income countries with a high number of residents without affordable health insurance and health care coverage, such as the United States, they have not acquired a role of health services providers [2]. Likewise, in Finland pharmacy chains have drifted pharmacies away from their primary health care scope towards more commercially-oriented enterprises selling health and wellness products and cosmetics, which are more profitable than selling heavily regulated medicinal products.

Even though not investigated in our study, policy-making can perform as a factor contributing to the dilemma that we found concerning pharmacy owners’ strategic commitment to health service orientation, while actual business is being driven by active product marketing [18]. This is because national incentives for long-term development of health-oriented services has been lacking, leading to more commercial strategic decisions at the individual pharmacy level in order to assure profits. Legislation and governmental policy papers may define health-oriented tasks for community pharmacies, but pharmacies’ financial ability to support this shift, which requires long-term development, has not been included in these policies, and has hardly been officially discussed [14, 23]. More should be learnt from countries that have succeeded to integrate community pharmacy services in their public health policies and strategies, such as the National Health Service (NHS) of the United Kingdom [2]. Further, more research should be available in order to acquire better understanding of the mechanisms leading to enhanced coordination and community pharmacists’ involvement in patient care and effectiveness as well as the economic impact of their involvement in care outcomes, to support large-scale implementation of these services [24–26].

Implementation of novel health-oriented services is a long process that requires strategic commitment and resource allocation [27]. Examples from other countries indicate that pharmacies can strengthen their professional and societal role by specializing in health services supporting safe and rational medication use [28]. It is still possible for independent, smaller community pharmacies to compete successfully following a differentiation or focused differentiation strategy which is well executed [3]. Finnish community pharmacies have particularly invested in improving medication counseling services since the 1990s, and more recently improved community pharmacists’ involvement in collaborative medication reviews [8, 24, 29]. The commitment of individual pharmacy owners to shared national health and medicines policy goals has been important. Finnish pharmacies’ polarization to two categories is evident: those basing their business on traditional dispensing and active product sales, and those willing to extend their operations towards professional health-oriented services. The situation is also similar in other countries [19].

The major limitation of this national study is the low response rate (34%), thus the results can only be considered as indicative. The response rate is similar to other recent email surveys targeting community pharmacists and pharmacy owners in Finland [30]. The geographic location, gender distribution and prescription volume of the respondents’ pharmacies represented all pharmacies in Finland well. It is possible that pharmacy owners who are interested in health service orientation and product marketing strategy work have been more active in responding than others [13]. Another limitation may be that the data is 7 years old and perhaps the practice has changed since data collection. The ongoing social and health services reform in Finland has shifted the political focus towards health service orientation after the survey data was acquired [23]. There would be a need for further research on the discrepancy between strategic health service orientation and actual service provision when discussing the future of pharmacies. More research is needed to understand root causes that hinder the larger-scale implementation of healthcare-oriented services in routine community pharmacy practice while strengthening the focus on active product sales. The contradiction that exists between pharmaceutical policy goals and the generation of income of pharmacies by providing health-oriented services should be examined as a contributing factor in future studies.

## Conclusions

Large pharmacies located close to rivals and belonging to marketing chains of individual community pharmacies differentiated as those having a high product marketing orientation. The health service orientation was not influenced by any of the explanatory variables used in this study. The discrepancy between high health service orientation scores and low actual service provision scores needs further investigation. The contradiction that exists between pharmaceutical policy goals and the generation of income of pharmacies should be examined as a contributing factor in this respect.

## Supplementary information

**Additional file 1.** Questionnaire, English translated version of the e-mail survey

**Additional file 2 Appendix 1.** Construction of the sum scales for health service orientation (13 variables) and active product marketing orientation (8 variables) of Finnish community pharmacies at the time of the survey: list of variables used in the sum scales. **Appendix 2.** Health-related services available in Finnish community pharmacies or part of the pharmacies at the time of the survey in 2013 that were included in the survey instrument. The services are presented in the order of their launch in Finland.

## Data Availability

The findings of this study are based on a national cross-sectional e-mail survey sent to privately owned community pharmacies in Finland. The data is not publicly available but can be accessed via the corresponding author.

## Abbreviations

ADD: Automated dose dispensing
CMR: Comprehensive medication review
COPD: Chronic obstructive pulmonary disease
NHS: National Health Service

## References

[1] Roberts A, Benrimoj S, Chen T, Williams K, Hopp T, Aslani P (2005). Understanding practice change in community pharmacy: a qualitative study in Australia. Res Soc Admin Pharm.

[2] Stubbings J, Nutescu E, Durley SF, Bauman JL (2011). Payment for clinical pharmacy services revisited. Pharmacotherapy.

[3] Singleton J, Nissen L. Future-proofing the pharmacy profession in a hypercompetitive market. Available online 29 June 2013, ISSN 1551-7411, 10.1016/j.sapharm.2013.05.010. 10.1016/j.sapharm.2013.05.010 23820045

[4] Wisell K (2019). The liberalization experiment. Understanding the political rationales leading to change in pharmacy policy. Digital Comprehensive Summaries of Uppsala Dissertations from the Faculty of Pharmacy.

[5] Bulajeva A, Labberton L, Leikola S, Pohjanoksa-Mäntylä M, Geurts MM, de Gier JJ, Airaksinen M (2014). Medication review practices in European countries. Res Social Adm Pharm.

[6] Reinikainen L, Lämsä E, Happonen P, Hämeen-Anttila K. Finnish Medicines Agency Fimea. Deregulation of the pharmacy system in Europe – actions and effects: Serial Publication Fimea Develops, Assesses and Informs 3/2017. ISBN 978-952-5624-75-5.

[7] FIP/WHO. Good Pharmacy Practise, 2011. Accessed 8.4.2020: http://www.fip.org/good_pharmacy_practice.

[8] Leikola S. Development and application of comprehensive medication review procedure to community-dwelling elderly. Doctoral dissertation. University of Helsinki, 2012. http://urn.fi/URN:ISBN:978-952-10-7698-5. Accessed Dec 13, 2016.

[9] Smith M, Cannon-Brel ML, Spiggle S. Consumer, physician, and payer perspectives on primary care medication management services with a shared resource pharmacists’ network. 2013, Available online 19 September ISSN 1551-7411. 10.1016/j.sapharm.2013.08.003. 10.1016/j.sapharm.2013.08.003 24055137

[10] Schommer J, Yusuf A, Hadsall R. Market dynamics of community pharmacies in Minnesota, US from 1992 through 2012. Available online 10 May 2013, ISSN 1551-7411. 10.1016/j.sapharm.2013.03.006. 10.1016/j.sapharm.2013.03.006 23669250

[11] Benrimoj SI, Frommer MS (2004). Community pharmacy in Australia. Aust Health Rev.

[12] Ym M, Mossialos E, Huseyin N, Courtin E (2013). Expanding the role of community pharmacists: Policymaking in the absence of policy-relevant evidence?. Health Pol.

[13] Association of Finnish Pharmacies. Annual Review 2012. http://www.apteekkariliitto.fi/media/pdf/afp_annual_review_2012_web.pdf. Accessed 12 Dec 2019.

[14] Ministry of Social Affairs and Health. Medicines Policy 2020. Publications of the Ministry of Social Affairs and Health 2011. https://www.julkari.fi/bitstream/handle/10024/111993/URN%3ANBN%3Afi-fe201504226140.pdf?sequence=1. Accessed Dec 13, 2016.

[15] Ministry of Social Affairs and Health (2015). Apteekkitoiminnan ja muun lääkehuollon kehittäminen. Final report. Reports and Memorandums 2015:4, [in Finnish] Helsinki.

[16] Ministry of Social Affairs and Health (2013). Lääkekorvausjärjestelmän kehittäminen. Lääkekorvausjärjestelmän kehittämistyöryhmän loppuraportti, STM:n julkaisu, [in Finnish] Helsinki.

[17] Association of Finnish Pharmacies (2011). The strategy and strategy goals 2012–2020. [in Finnish] Helsinki.

[18] Jokinen L, Puumalainen I, Airaksinen M (2019). Influence of strategic planning on product marketing and health service orientation of community pharmacies - a national survey in Finland. Health Policy.

[19] Imfeld-Isenegger T, Soares IB, Makovec UN (2020). Community pharmacist-led medication review procedures across Europe: Characterization, implementation and remuneration. Res Social Adm Pharm.

[20] Dawoud D, Haines A, Wonderling D, Ashe J, Hill J, Varia M, Dyer P, Bion J (2019). Cost effectiveness of advanced pharmacy services provided in the community and primary care settings: a systematic review. Pharmacoeconomics.

[21] Houle S, Carter C, Tsuyuki R, Grinrod K (2019). Remunerated patient care services and injectons by pharmacist: an international update.

[22] Vogler S, Habimana K, Arts D (2014). Does deregulation in community pharmacy impact accessibility of medicines, quality of pharmacy services and costs?Evidence from nine European countries. Health Pol.

[23] Ministry of Social Affairs and Health (2018). Rational Pharmacotherapy Action Plan. Reports and Memorandums of the Ministry of Social Affairs and Health.

[24] Toivo T, Dimitrow M, Puustinen J, Savela E, Pelkonen K, Kiuru V, Suominen T, Kinnunen S, Uunimäki M, Kivelä SL, Leikola S, Airaksinen M (2018). Coordinating resources for prospective medication risk management of older home care clients in primary care: procedure development and RCT study design for demonstrating its effectiveness. BMC Geriatr.

[25] Kallio S, Kiiski A, Airaksinen M, Mäntylä A, Kumpusalo-Vauhkonen A, Järvensivu T, Pohjanoksa-Mäntylä M (2018). Community Pharmacists' contribution to medication reviews for older adults: a systematic review. J Am Geriatr Soc.

[26] Kallio S, Kumpusalo-Vauhkonen A, Järvensivu T, Mäntylä A, Pohjanoksa-Mäntylä M, Airaksinen M (2016). Towards interprofessional networking in medication management of the aged: current challenges and potential solutions in Finland. Scand J Prim Health Care.

[27] White L, Klinner C (2012). Service quality in community pharmacy: an exploration of determinants. Res Soc Admin Pharm.

[28] Eades C, Ferguson J, O'Carroll R (2011). Public health in community pharmacy: a systematic review of pharmacist and consumer views. BMC Public Health.

[29] Puumalainen I (2005). Development of instruments to measure the quality of patient counselling. Doctoral dissertation.

[30] Westerling A (2011). Information technology development needs in community pharmacies.

[31] Kurko T. Deregulation of nicotine replacement therapy products in Finland: reasons for pharmaceutical policy changes and reflections on smoking cessation practices. Doctoral dissertation, University of Helsinki 2015. http://urn.fi/URN:ISBN:978-951-51-1223-1. Accessed Jan 13, 2019.

[32] Ministry of Social Affairs and Health (2011). Avohuollon apteekkitoiminnan kehittämistarpeet. Sosiaali- ja terveysministeriön selvityksiä 2011:16, [in Finnish]. Helsinki Finland.

[33] Sinnemäki J, Sihvo S, Isojärvi J, Blom M, Airaksinen M, Mäntylä A (2013). Automated dose dispensing service for primary healthcare patients: a systematic review. Syst Rev.

