# Supplementary material for: Factors associated with health service orientation and active product marketing orientation in Finnish community pharmacies: a nationwide study among private pharmacy owners
Source: BMC Health Serv Res. 2020 Jul 20;20:667. doi: 10.1186/s12913-020-05469-y (PMC7370436; doi:10.1186/s12913-020-05469-y)
Supplement: Supplementary file 1 — Additional file 1. Questionnaire, English translated version of the e-mail survey [file 12913_2020_5469_MOESM1_ESM.pdf]

National pharmacy development survey

The purpose of this nationwide survey is to survey pharmacists' views on the need to develop their pharmacy business. The results of this survey will be used as part of research at the department of Social Pharmacy, University of Helsinki.

The responces are transferred to the researcher anonymously and the data of the individual respondents cannot be identified at any stage of the research.

BACKGROUND QUESTIONS

Location by province

- o Southern Finland
- o Western Finland
- o Eastern Finland
- o Northern Finland

Location area

- o Countryside
- o City, center
- o City, suburb
- o City, commercial center

Annual prescription volume

- o Less than 40 000
- o 40 000-60 000
- o 60 001-100 000
- o More than 100 000

Work experience as a pharmacy owner

- o Less than 5 years
- o 5-10 years
- o More than 10 years

Pharmacy owner's gender

- o Female
- o Male

CURRENT SERVICES

Does your pharmacy offer the following health-oriented services?

	yes	no
Health check service	o	o
Smoking cessation	o	o
Services for special groups	o	o
Medication consultation	o	o
Inhaler technique check service	o	o
Comprehensive medication review	o	o
Medication review	o	o

Health measuring e.g. blood pressure	o	o
Automated dose dispensing	o	o

MARKETING

What do you think about the following statements about your own situation? If you wish, you can comment on the answer on the side of your answer column.

	totally agree	partly agree	partly disagree	totally disagree	I can't say
The pharmacy monitors the success of campaigns	o	o	o	o	o
The pharmacy has a person responsible for campaigns or product marketing	o	o	o	o	o
Pharmacy chains have growing importance in the future	o	o	o	o	o
Product marketing efforts will increase in pharmacies	o	o	o	o	o
It is necessary for pharmacies to expand product portfolio to ensure the economy	o	o	o	o	o
The pharmacy favors free trade brands that are sold only in pharmacies	o	o	o	o	o
The pharmacy is actively investing in additional sales	o	o	o	o	o

PHARMACEUTICAL SERVICE PRODUCTION AND SUPPLEMENTARY PROFESSIONAL SERVICES

What do you think about the following statements about your own situation? If you wish, you can comment on the answer on the side of your answer column.

	totally agree	partly agree	partly disagree	totally disagree	I can't say
Pharmacies should commercialize services	o	o	o	o	o
Future pharmacy activities will orient more towards health promotion services	o	o	o	o	o
My pharmacy is interested in new paid services that improve medication safety	o	o	o	o	o
My pharmacy is interested in new services such as inhalation check for asthma patiens	o	o	o	o	o
My pharmacy is interested in developing online pharmacy services	o	o	o	o	o
My pharmacy is interested in providing paid services such as telephone counselling by the pharmacist	o	o	o	o	o

Development of new services is important for pharmacy business	o	o	o	o	o
Pharmacy service points will assure access to medicines in sparsely populated areas in the future	o	o	o	o	o
The online pharmacy services will extend to operations of nearly every pharmacy	o	o	o	o	o
Community pharmacies could also provide vaccination services	o	o	o	o	o
Pharmacies are willing to increase specific competencies required for specific services (e.g., comprehensive medication reviews)	o	o	o	o	o
Comprehensive medication review services will be an important part of future activities of community pharmacies	o	o	o	o	o
Pharmacies could become substitutes for primary health care units in areas with no public health centers	o	o	o	o	o
