# Supplementary material for: Factors associated with health service orientation and active product marketing orientation in Finnish community pharmacies: a nationwide study among private pharmacy owners
Source: BMC Health Serv Res. 2020 Jul 20;20:667. doi: 10.1186/s12913-020-05469-y (PMC7370436; doi:10.1186/s12913-020-05469-y)
Supplement: Supplementary file 2 — Additional file 2 Appendix 1. Construction of the sum scales for health service orientation (13 variables) and active product marketing orientation (8 variables) of Finnish community pharmacies at the time of the survey: list of variables used in the sum scales. Appendix 2. Health-related services available in Finnish community pharmacies or part of the pharmacies at the time of the survey in 2013 that were included in the survey instrument. The services are presented in the order of their launch in Finland. [file 12913_2020_5469_MOESM2_ESM.docx]

Additional file 2: **Appendix 1.** Construction of the sum scales for health service orientation (13 variables) and active product marketing orientation (8 variables) of Finnish community pharmacies at the time of the survey: list of variables used in the sum scales.

Variables (n=13) used to operationalize health services orientation of Finnish community pharmacies. The internal consistency of the variables, calculated by using reliability analysis (Cronbach’s Alpha), was 0.836. A 5-point Likert-scale was applied for responses.

| Pharmacy service points will assure access to medicines in sparsely populated areas in the future |
| --- |
| The online pharmacy services will extend to operations of nearly every pharmacy service |
| Future pharmacy activities will orient more towards health promoting services |
| My pharmacy is interested in providing paid services such as telephone counselling by the pharmacist |
| Community pharmacies could also provide vaccination services |
| My pharmacy is interested in new paid services that improve medication safety |
| Comprehensive medication review services will be an important part of the future activities of community pharmacies |
| Pharmacies could become substitutes for primary health care units in areas with no public health centers |
| My pharmacy is interested in developing online pharmacy services |
| My pharmacy is interested in new services such as inhalation checks for asthma patients |
| Development of new services is important for pharmacy business |
| Pharmacies should commercialize services |
| Pharmacists are willing to increase specific competencies required for specific services (e.g., comprehensive medication reviews) |

Variables (n=8) used to operationalize the product marketing orientation of Finnish community pharmacies. The internal consistency of the variables, calculated by using reliability analysis (Cronbach’s Alpha), was 0.699. A 5-point Likert-scale was applied for responses.

| Focus on product marketing is an important part of the strategy |
| --- |
| Pharmacy chains have a growing importance in the future |
| Product marketing efforts will increase in pharmacies |
| The pharmacy is actively investing in additional sales |
| It is necessary for pharmacies to expand their product portfolio to ensure their economic success |
| The pharmacy favors free trade brands that are sold only in pharmacies |
| The pharmacy monitors the success of campaigns |
| The pharmacy has a person responsible for campaigns or product marketing |

**Appendix 2.** Health-related services available in Finnish community pharmacies or part of the pharmacies at the time of the survey in 2013 that were included in the survey instrument. The services are presented in the order of their launch in Finland.

| **Service** | **Description of the service** | **Developer and year of implementation** |
| --- | --- | --- |
| Automated dose dispensing (ADD) | A service in which a pharmacy dispenses client’s regularly used medicines (two weeks at a time) as machine-packed unit doses for each time of administration. ADD is recommended to Include medication review.^31-33^ | The Association of Finnish Pharmacies, 2002 |
| Comprehensive medication review (CMR) | The procedure is for a comprehensive  clinical review of all medications used. It is based on collaboration between  pharmacists and other health care professionals, particularly physicians, and includes  access to clinical patient data, a home visit with a patient interview, a written case report with DRPs and recommended actions to solve them, a case conference with the physician and a follow up of implementation of the recommended changes in the medication. Requires accreditation training for pharmacists. ^8,31^ | The National Coordination Group concerning new professional community pharmacy services, 2005 |
| Smoking cessation | A service based on individual support and counseling that aims to assist in the quitting of smoking using NRT products. ^31^ | The Association of Finnish Pharmacies, 2005 |
| Asthma/diabetes service | Enhanced medication counseling service for specific patient groups. Requires accreditation training for pharmacists to perform. | The Association of Finnish Pharmacies, 2009 |
| Medication consultation | Consulting service to assist social and healthcare units to establish their plan for safe medication practices. | The Association of Finnish Pharmacies, 2011 |
| Inhalation technique check-up service for asthma and chronic obstructive pulmonary  disease (COPD) patients | The service is targeted to those asthma and COPD patients who may have problems with taking their inhaled medications. Another target group is children and adolescents at the point of starting to take responsibility for their medication | The Association of Finnish Pharmacies, 2012 |
| Health check | A service for assessing client’s lifestyle-related health risks and the most common clinical measures for estimating health risks, such as heart attack, stroke and diabetes | The Association of Finnish Pharmacies, 2012 |
| Medication review/prescription review | A service for reviewing the client’s medication list. The purpose is to identify duplicate medications, inappropriate dosing and drug-drug interactions. ^31^ | The Association of Finnish Pharmacies, 2012 |
